# Decolourisation of triphenylmethane dyes by biogenically synthesised iron nanoparticles from fungal extract

**DOI:** 10.1080/21501203.2021.1948928

**Published:** 2021-07-17

**Authors:** Simon Schuster, Adeline Su Yien Ting

**Affiliations:** a School of Science, Monash University Malaysia, Jalan Lagoon Selatan, Malaysia; b Hochschule Esslingen, Esslingen Am Neckar, Germany

**Keywords:** Decolourisation, endophyte extract, iron nanoparticles, *Fusarium* sp, triphenylmethane dyes

## Abstract

In this study, the extract from endophytic *Fusarium proliferatum* was used to synthesise iron nanoparticles (Fe-NPs). The properties of the biogenically synthesised Fe-NPs were then characterised by field emission scanning electron microscopy (FE-SEM), energy-dispersive X-ray spectroscopy (EDX) and Fourier transform infrared spectroscopy (FTIR). The efficacy of the synthesised Fe-NPs in decolourizing triphenylmethane dyes was evaluated. Results revealed that fungal extract from *F. proliferatum* was successfully used to synthesise Fe-NPs. The Fe-NPs produced were 20–50 nm in size, and consist of substantial elemental Fe content (14.83%). The FTIR spectra revealed the presence of amino acids and proteins on the surface of the Fe-NPs, confirming the biogenic synthesis of the Fe-NPs. When tested for decolourisation, the Fe-NPs were most effective in decolourising Methyl Violet (28.9%), followed by Crystal Violet (23.8%) and Malachite Green (18.3%). This study is the first few to report the biogenic synthesis of Fe-NPs using extracts from an endophytic *Fusarium* species and their corresponding dye decolourisation activities.

## Introduction

Nanotechnology has become an important field of research in recent years. Nano-sized particles (between 1 and 100 nm in size) have high surface-to-volume ratio. As such, they enhance catalytic activities, which makes them highly important for microbiological and biotechnological applications (Christian et al. 2008). Examples of nanoparticles with various applications include metal nanoparticles such as gold, platinum, iron, nickel, and copper. Gold (Au-NPs) and platinum (Pt-NPs) nanoparticles have been used for biomedical applications (Hrapovic et al. 2004; Sperling et al. 2008) while iron nanoparticles (Fe-NPs) are useful for medical applications, as well as for dye removal and antibacterial applications (Pankhurst et al. 2003; Radini et al. 2018).

Some nanoparticles are reportedly able to enhance catalytic processes, which improves degradation of pollutants, while some are useful for the synthesis of active molecules. Nickel-iron oxide nanoparticles (Ni/Fe_3_O_4_-NPs) and copper oxide nanoparticles (CuO-NPs) enhance degradation of organic dye pollutants when used in combination with UV-irradiation (Pakzad et al. 2019). These metal nanoparticles are also able to remove antibiotics found in wastewaters such as metronidazole, ciprofloxacin and cephalexin (Pakzad et al. 2020). Nanoparticles can also be used to immobilise chemical complexes to aid in reducing pollutants. Copper(II)-5-phenyl-1 H-tetrazole complex immobilised on the surface of Fe_3_O_4_/SiO_2_-NP, functions as a catalyst with the reducing agent NaBH_4_ to degrade 4-nitrophenol (4-NP), 2,4-dinitrophenylhydrazine (2,4-DNPH), methylene blue (MB) and Nigrosin (NS) (Nasrollahzadeh et al. 2018). In addition, nanoparticles are also effective for the synthesis of active molecules. Au-NPs, CuO-NPs and Pd/Fe_3_O_4_-NPs have been used to catalyse the formation of propargylamine (Nasrollahzadeh and Sajadi 2015; Nasrollahzadeh et al. 2015a), N-arylation of nitrogen-containing heterocycles (Nasrollahzadeh et al. 2015b), and the amination of aldehydes at room temperature (Nasrollahzadeh and Sajadi 2016), respectively.

The conventional approach to synthesise metal nanoparticles is via physical techniques such as laser ablation (Mafuné et al. 2001) or chemical approaches using chemical-based reducing agents (Chen et al. 2001). These approaches are relatively expensive and toxic, owing to the chemicals used (i.e. reducing agents, solvents) which are hazardous to the environment. To mitigate this, biological alternatives are considered in which the biological-based agents are used to substitute the chemical reagents, reducing risks and hazards. Various biological extracts have been investigated as possible biological agents to synthesise metal NPs. They include extracts from plants such as *Euphorbia polygonifolia* (Pakzad et al. 2020), *E. maculata* (Pakzad et al. 2019), *E. stracheyi* Boiss (Nasrollahzadeh and Sajadi 2016) and *E. condylocarpa* M. bieb (Nasrollahzadeh et al. 2014). Other plants such as *Tamarix gallica* (Nasrollahzadeh et al. 2015b) and *Anthemis xylopoda*, have also been studied, particularly the use of the leaf and flower extracts (Nasrollahzadeh and Sajadi 2015; Nasrollahzadeh et al. 2015a). Biological extracts were also obtained from microorganisms, such as bacteria (Roh et al. 2001), actinomycetes (Ahmad et al. 2003), yeast (Kowshik et al. 2002) and fungus (Mukherjee et al. 2002). The diverse biological resources used typically result in nanoparticles with a wide range of sizes, shapes, compositions, and physicochemical properties (Mohanpuria et al. 2008).

The capacity of fungal extracts in synthesising metal nanoparticles is attributed to the constituents found in the fungal extract. Fungal extracts are rich in proteins and enzymes, which are effective reducing agents (Bharde et al. 2006; Narayanan and Sakthivel 2010). Fungal extracts also consist of metabolites, amino acids, vitamins, polysaccharides, organic acids, as well as phenolics (Iravani 2011; Luo et al. 2014). The presence of these constituents contributes to the reducing and capping effect on the metal solutions to produce metal nanoparticles, mimicking the role of chemical reagents. Several fungal isolates have been used for the synthesis of NPs, with a common observation that various fungal species synthesise NPs of different sizes. This includes *Pochonia chlamydosporium, Aspergillus fumigatus, Aspergillus wentii, Ceroprepes lunata* and *Chaetomium globosum*. These isolates produce extracts that were able to form Fe-NPs with a size range of 5–200 nm (Kaul et al. 2012). Some distinct differences were observed though, with extracts from *Alternaria alternata* yielding 9 nm-sized NPs (Mohamed et al. 2015), while endophytic *Fusarium oxysporum* and *Verticillium sp*. produced spherical Fe-NPs of 20–50 nm sizes (Bharde et al. 2006).

In this study, the extract from a fungal endophyte isolated from a medicinal plant is used. Endophytes are microorganisms that exist inside host plants. They have been known to produce a range of bioactive compounds such as antimicrobial, antioxidant, or antitumor compounds (Ting et al. 2021). It has long been hypothesised that endophytes may evolve to produce similar bioactive compounds as their host plants. In this study, the endophyte used, a *Fusarium* sp., was isolated from a medicinal plant *Cymbopogon citratus*. The endophyte was selected as it is expected to produce a range of bioactive compounds similar to compounds in the host plant, which then may be an ideal bioagent to synthesise the NPs.

Of the various metal nanoparticles, Fe-NPs are valuable as they have a broad range of applications. Fe-NPs showed antibacterial activity against gram negative *Pseudomonas aeruginosa* (Senthil et al. 2012). Fe-NPs have also been used for wastewater treatment to remove heavy metals (Tandon et al. 2013) and trichloroethylene (He and Zhao 2005), as well as for decolourisation of malachite green (MG) dye (Weng et al. 2013). Metal nanoparticles, however, have not been explored for their efficacy in decolourising other common triphenylmethane (TPM) dyes such as methyl violet (MV), crystal violet (CV) and cotton blue (CB), which are widely used in the textile and pharmaceutical industries (Saratale et al. 2006; Jasińska et al. 2012). Due to their chemical nature, TPM dyes are resistant to natural degradation processes (Przystaś et al. 2012). As they are mutagenic and toxic to living organisms, it is pertinent that they are removed from wastewaters (Saratale et al. 2006).

This study aims to determine the functional role of the fungal extract of *F. proliferatum* in synthesising Fe-NPs. This approach provides for a cheaper and greener alternative to existing methods. The characteristics of Fe-NPs produced are profiled, and their efficacies in decolourising TPM dyes are also established.

## Materials and methods

### Molecular typing of the endophyte

Endophyte isolate CCH was obtained as an established pure culture on Potato Dextrose Agar (PDA, Merck). It was isolated as an endophyte from a medicinal plant, *Cymbopogon citratus* (Chow and Ting 2015), and was kept as silica-gel stock culture since the last 5 years. As such, the molecular typing of the isolate was performed to validate the species of the isolate. The isolate was identified via partial DNA-sequencing of the 18S rRNA. To initiate fungal culture, mycelial plugs were transferred onto PDA and sub-cultured for 7 days (30 ± 2°C). The mycelia (20 mg wet weight) were then scraped from the agar plate and ground in liquid nitrogen. The GF-1 Plant DNA extraction kit (Vivantis Technologies, USA) was used to extract the genomic DNA. PCR amplification of the internal transcribed spacer (ITS1, 5.8S rRNA, ITS2) gene region was conducted using universal primer pairs of ITS 1 (5‘-TCCGTAGGTGAACCTGCGG-3‘) and ITS 4 (5‘-TCCTCCGCTTATTGATA TGC-3‘) (Chen and Yien Ting 2015; Chow and Ting 2015). The collection and purification of genomic DNA was performed with the MEGAqucik-spin™ plus Fragment DNA Purification Kit (iNtRON Biotechnology, Korea), prior to sequencing by 1st Base (Malaysia). For identification of isolate CCH the NCBI BLAST tool was used (https://blast.ncbi.nlm.nih.gov/Blast.cgi).

### Harvesting fungal extracts

To obtain fungal extracts, seven mycelial plugs were inoculated into 100 mL PDB (Difco Potato Dextrose Broth, BD), and incubated as standing culture for 10 days (25 ± 2°C). After incubation, the fungal biomass was collected through filtration with double-layer cheesecloth and washed thrice with SDW (sterile distilled water) to remove media residues. The biomass obtained was further incubated in Milli-Q water (Milli-Q, Merck) at a final concentration of 100 g of biomass/L and incubated for 1 day at room temperature (RT, 24 ± 2°C). After incubation, the mixture (biomass-solution) was then boiled for 30 min to enhance extraction of fungal extracts. The mixture was then filtered once more with double-layer cheesecloth. The filtrate collected was used for the synthesis of Fe-NPs.

### Synthesis of Fe-NPs with fungal extracts

The Fe-NPs were synthesised based on methods by Rajendran and Sen (2016), with slight modifications to the precursor solution and concentration used, pH, temperature, and mixture ratio. In this study, a precursor solution (50 mL), containing 5 mM FeCl_3_ (R&M Chemicals) and 5 mM FeSO_4_ (Riendemann Schmidt), was first prepared and the pH adjusted to 7. The fungal extract was then mixed with the precursor solution (in a 500 mL-flask) in a ratio of 2:1 of extract: precursor solution, and incubated with agitation (350 rpm) for 24 h at 35 ± 2°C. After incubation, the mixture, now containing Fe-NPs, was centrifuged for 10 min at 5000 rpm and the supernatant discarded. The Fe-NPs were washed with generous amount of Milli-Q water, centrifuged and the supernatant discarded again. The Fe-NPs collected were then spread onto a filter-paper and dried at 45°C for 2 days in an oven. A separate control set was prepared for the experiment, using a mixture of Milli-Q water (instead of fungal extract) and precursor solution (same ratio and incubation conditions). This control set will detect if the iron salts form particles on its own, without the mixture with fungal extracts.

### Characterisation of Fe-NPs

Ultraviolet-visible (UV-vis) spectra in the range of 200–600 nm were read with the UV-vis spectrophotometer (Lambda 365, Perkin Elmer) for the following samples; the fungal extract, the precursor solution (in precipitate form) and the synthesised Fe-NPs. Assessments were made at different time points. Peaks at wavelengths of 260–280 nm indicates the presence of biological constituents such as amino acids in the extract, which may be involved in the synthesis of Fe-NPs (Prasad et al. 2017). These constituents aid as capping and reducing agents in the formation of Fe-NPs.

Field Emission Scanning Electron Microscopy (FE-SEM) in combination with Energy Dispersive X-ray spectroscopy (EDX) was performed to analyse the surface appearance, size, as well as the elemental composition of the synthesised Fe-NPs and the precursor precipitates (Chin and Ting et al. 2020). The dried Fe-NPs and precursor precipitates (both prepared in powder form) were sputter-coated with platinum for 38 s at 30 mA (Quorum; Q150RS) and analysed (Hitachi, SU8010; Oxford-Horiba, Inca XMax50 EDX). The detection of spherical-shaped Fe-NPs, with a diameter between 20 and 100 nm, indicates successful formation of Fe-NPs (Bharde et al. 2006). The presence of the elements, i.e. carbon, oxygen and phosphorous, indicate the formation and capping of Fe-NPs with organic compounds.

Functional groups on the surface of Fe-NPs and precursor precipitates were identified with FTIR analysis (Perkin Elmer, Spectrum Two). This was analysed by performing the attenuated total reflection spectra of the powder forms of Fe-NPs and the precursor precipitate, by scanning the mid-infrared region from 4000 to 400 cm^−1^. These functional groups can be related to the organic constituents from the fungal extract, which are identified as reducing and capping agents for the formation of Fe-NPs (Mohan Kumar et al. 2013).

### Dye decolourisation assay

Dye solutions, i.e. Malachite Green (Riendemann Schmidt), Methyl Violet (Fluka Analytical), Crystal Violet (Merck) and Cotton Blue (Sigma Life Science), were freshly prepared from powdered dyes at a concentration of 100 mg/L in Milli-Q water (Chen and Yien Ting 2015). The dye solutions (10 mL) were then dispensed into 15 mL Falcon tubes, followed by the addition of 5 mg of powdered Fe-NPs. Two control sets were prepared. The first set has a similar set-up, substituting Fe-NPs with precursor precipitates, while the other control set involved untreated dye solutions. All tubes were incubated with agitation (150 rpm), at 24 ± 2°C in the dark. At every 24 h interval, 100 µL of dye solution was pipetted from each tube into fresh centrifuge tubes and centrifuged (10,000 rpm, 10 min). The supernatant obtained was diluted with Milli-Q water in 96-well plates (Jet Biofil®). The absorbance was read at the respective wavelengths for each dye: 617 nm (MG), 584 nm (MV), 590 nm (CV) and 599 nm (CB) (Tecan®, Infinite M200 Plate Reader) (Chen and Yien Ting 2015). The absorbance values were recorded and the decolourization efficiency (DE, %) (Parshetti et al. 2006) calculated as in Equation (Eq.1).

(Eq. 1) $$DE\left(\%\right)=\frac{\begin{array}{c} InitialAbsorbance\\ -Absorbanceaftertreatment \end{array}}{Initialabsorbance}\times 100\%$$

Additionally, the UV-vis spectra of the samples within the range of 300–800 nm were also recorded. The decrease of a peak indicates decolourization of dye molecules (Ting et al. 2016).

### Statistical analysis

All experiments were performed in triplicates. Data collected were analysed with Analysis of Variance (ANOVA) and the means compared using the Tukey–Kramer multiple comparison test (honestly significant difference (HSD), P < 0.05) or paired T-test (P < 0.05 for paired comparisons) where relevant (Ting et al. 2016).

## Results and discussion

### Taxonomic identification of isolate CCH

The DNA sequencing results of the ITS region of isolate CCH indicated 99.48% homology (E value of 0.0, query coverage of 99%) with *Fusarium proliferatum* isolate CCH (MK685139.1). This sequence has been deposited in the NCBI and this confirms the species of the isolate, although in storage for a prolonged period of time. The complete BLAST result is provided as a Supplementary file.

### Synthesis of Fe-NPs with extract from endophytic F. proliferatum

**Figure 1.**
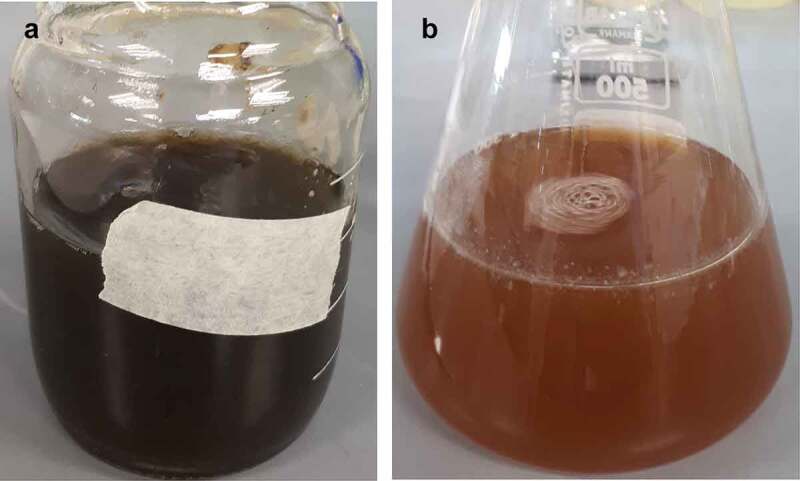
(A) Precursor solution containing 5 mM FeCl_3_ and 5 mM FeSO_4_ at pH 7, and (B) after mixing with the extract of *F. proliferatum*. Dark greenish colour indicates the presence of oxidised forms of iron, while the colour change to orange/brown is attributed to the formation of reduced forms of iron.

The mixing of extract of *F. proliferatum* with the precursor solution (containing FeCl_3_ and FeSO_4_) resulted in the immediate change of colour of the mixture from greenish brown/black to orange/brown colour (Figure 1). This was attributed to the formation of reduced forms of Fe. The change in colour of the mixture has also been reported by other researchers and is deemed relatively typical for the synthesis of Fe-NPs with extracts (Radini et al. 2018). Upon closer examination, small particulates (Fe-NPs) were observed suggesting the possible formation of Fe-NPs.

### Characterisation of Fe-NPs

**Figure 2.**
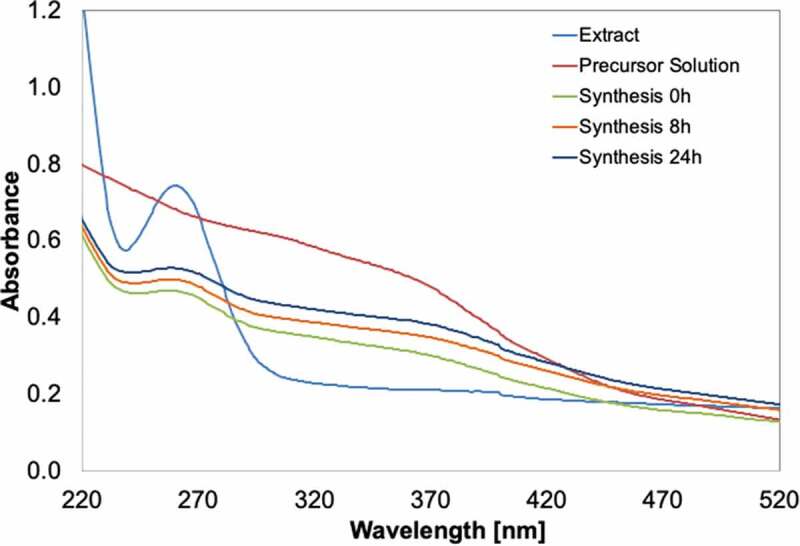
UV-vis spectra of the extract from *F. proliferatum*, the precursor salt solution (5 mM FeCl_3_ + 5 mM FeSO_4_, pH 7), and the Fe-NPs synthesised, sampled at various time intervals.

The synthesised Fe-NPs were found to demonstrate properties typical of a metal nanoparticle. Firstly, the Fe-NPs (synthesised at 0, 8, 24 h) showed a spectrum similar to the precursor solution (Figure 2). This affirms the integrity of the presence of Fe in the synthesised Fe-NPs. The Fe-NPs also have a slight peak detected at 260–270 nm, similar to the spectrum of the fungal extract. This peak reflects the presence of biological constituents such as protein or amino acids (e.g. tryptophan, tyrosine, phenylalanine). These amino acids have their maximum absorption at 280, 275 and 257 nm, respectively (Prasad et al. 2017). These constituents may have critical roles in the formation and stabilisation of Fe-NPs that were synthesised from the extract. These similar spectra peaks were also detected at 260–270 nm for fungal extract. The presence of similar peaks in both fungal extract and Fe-NPs indicated that the Fe-NPs were biogenically synthesised using the fungal extract (Figure 2). This peak is clearly absent from pre-cursor solutions, denoting the non-biological nature of the pre-cursor solution. The presence of spectra peaks of biological origin is therefore strong evidence of the successful biogenic synthesis of metal-NPs (Radini et al. 2018).

**Figure 3.**
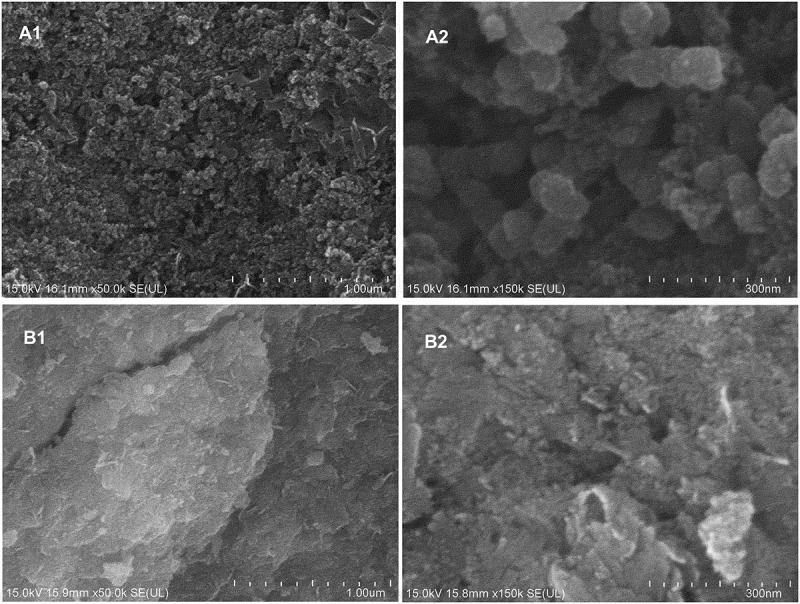
Scanning Electron Microscopy (SEM) micrographs of (A) Fe-NPs and (B) precursor precipitates at 50x (A1, B1) and 150x (A2, B2) magnification. Scale bar 1 µm/300 nm.

The synthesised Fe-NPs and precursor precipitates were analysed using FE-SEM and EDX, and results revealed notable differences in structures and compositions (Figure 3). Fe-NPs showed spherical structures with a diameter of 20–50 nm. The same properties have been discovered for Fe-NPs synthesised with *F. oxysporum* (Bharde et al. 2006). On the contrary, precursor precipitates had a larger, dense, plate-like surface, and spherical structures were absent. This showed that the Fe-NPs synthesised with extract of *F. proliferatum*, were able to give rise to nano-structured particles. It also highlighted the role of organic compounds of the extract, which provided effective reducing and capping effect, to produce spherical structures of the NPs. This was completely absent in metal pre-cursors (precursor precipitates) devoid of exposure to the fungal extract.

**Figure 4.**
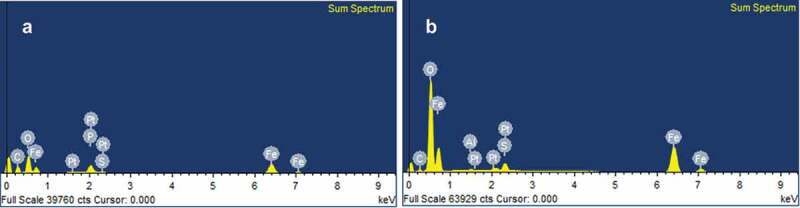
Electron-Dispersive X ray (EDX) spectra for (A) Fe-NPs and (B) precursor precipitates.

The EDX-results further demonstrated the difference between the synthesised particles with the precursor and the precursor precipitates. Fe-NPs had characteristically higher composition of carbon (41.28%), suggesting that carbon-based compounds from fungal extract may have been integrated into the Fe-NPs (Table 1, Figure 4). Phosphorous (P) was also detected, indicating the presence of organic constituents in the Fe-NPs. The presence of P may only be 3.65% in Fe-NPs but was evidently absent in precursor precipitates. This strongly suggested that the P element may have been derived from the amino acids and organic compounds found in the fungal extract. Fe was also detected in the Fe-NPs (14.83%) albeit at slightly lower levels than original Fe levels in the precursor precipitates (21.10%) (Table 1). This shows that biogenically synthesised Fe-NPs have a higher proportion of C, followed by lesser metal composition, and the presence of P, compared to precursor precipitates. This composition was found to be similar to Fe-NPs synthesised with green tea extract (Weng et al. 2013), as they contained 16.80% of Fe, 30.60% C and 34.76% O. They proposed that the high composition of C and O was attributed to organic compounds such as polyphenols of the green tea extract. For Fe-NPs synthesised with fungal extract, the high composition of carbon comes from amino acids, proteins and other organic compounds of the extract that aided in the synthesis of Fe-NPs. The most important role of proteins is that they are able to reduce iron and aid as capping agents to stabilise the Fe-NPs. This has been observed in the formation of different metal nanoparticles using fungal extracts from *Alternaria alternata* (Baharvandi et al. 2015; Sarkar and Acharya 2017) and other species (AAAj and Azzah 2015).

**Table 1.** Elemental composition of Fe-NPs and precursor precipitates determined by EDX-analysis

Percentage (%)	Fe	C	O	P	S	Pt
Fe-NPs	14.83	41.28	39.45	3.65	0.38	0.41
Precursor precipitates	21.10	6.97	69.32	-	2.03	0.32

- not detected.

**Figure 5.**
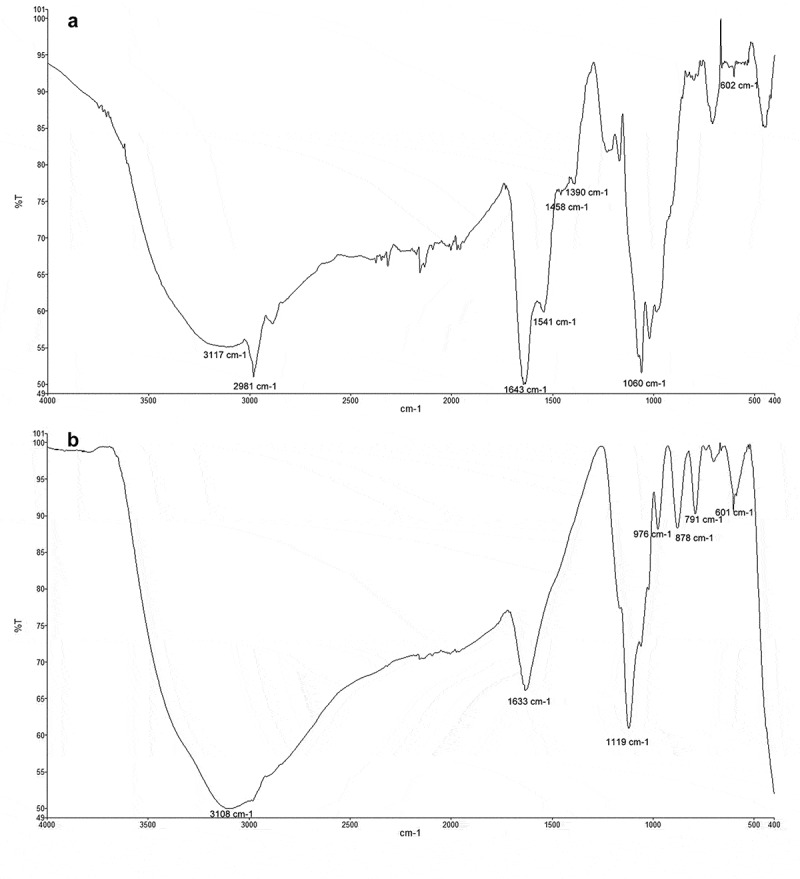
FTIR-spectra of powderised (A) Fe-NPs and (B) precursor precipitates.

The FTIR analysis revealed the presence of several main functional groups in Fe-NPs and the precursor precipitates. For Fe-NPs, several functional groups suggesting biological origin were detected. This includes various peaks at 3117 cm^−1^ (shows the O-H/N-H stretch vibration from carboxylic/amino acids), at 1643 cm^−1^ (for the C = O bond (amide-I)), at 1541 cm^−1^ (for the N-H bond (amide-II)), and at 1060 cm^−1^ (for the C-O bond of carboxylic acids) (Figure 5). These peaks indicated the presence of amino acids and proteins (Gurunathan et al. 2009; Correa-Llantén et al. 2013), which aided in stabilising the Fe-complex (Bharde et al. 2006; Radini et al. 2018). The peak at 2981 cm^−1^ showed the stretching vibration of the alkane carbon skeleton of the proteins, and the peaks at 1458 cm^−1^ and 1390 cm^−1^ showed the bend vibrations of -CH_3_. The Fe-O bond can be seen at 602 cm^−1^ in the spectra of Fe-NPs and precursor precipitates (Figure 5), proving the presence of iron (Bruni et al. 1999; Feng et al. 2008). This is the only peak found present in both spectra. For the precursor precipitates, peaks at 3108 (O-H from remaining water in the sample) 1633, 1119, 976, 878 and 791 cm^−1^ could be detected. The FTIR spectra revealed that organic compounds of the *F. proliferatum* extract, such as proteins and amino acids, are necessary as reducing agents. They are important in the formation of Fe-NPs influencing the size, shape and composition as the Fe-NPs. These functional groups on the surface of the Fe-NPs play a significant role as well in the dye decolourisation process of the TPM dyes. These structures (-OH groups or peptide bond from proteins) are able to adsorb the dye molecules through binding of the cationic dyes.

### Dye decolourisation assay

**Figure 6.**
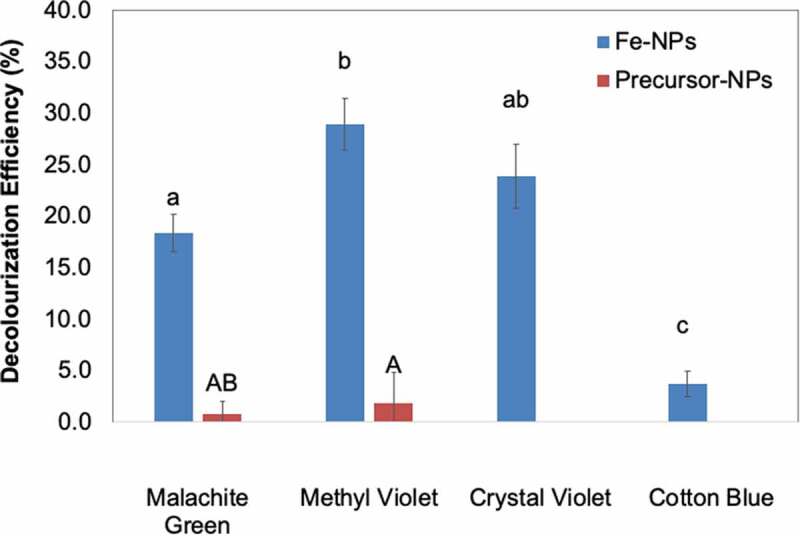
Decolourization Efficiency (DE, %) of Fe-NPs and precursor precipitates towards various TPM-dyes (MG-malachite green, MV-methyl violet, CV-crystal violet and CB-cotton blue). Means with the same letters and caption are not significantly different at HSD_(0. 05)_. Error bars indicate Standard error of mean (±SEM).

The decolourisation assay revealed that Fe-NPs were effective in decolourising three of the TPM dyes tested (MG, MV and CV) (Figure 6). The organic compounds in the fungal extract were not only necessary to synthesise Fe-NPs, but also in allowing effective dye decolourisation. The decolourisation efficiencies by Fe-NPs were 18.3% (MG), 28.9% (MV) and 23.8% (CV). As expected, the precursor precipitates did not decolourise any of the TPM dyes tested (Figure 6). The Fe-NPs were however, unable to decolourise CB, and this may be attributed to the high dye concentration, which saturated the surface of the Fe-NPs. CB is often tested at 50 mg/L, while other TPM dyes at a concentration of 100 mg/L (Ting et al. 2016; Munck et al. 2018). In this study, however, all dyes were prepared for assays at a concentration of 100 mg/L. Fe-NPs appeared to have higher affinity to remove MV compared to other TPM dyes, with 28.9% of MV removed at the end of 8 days (Figure 6).

**Figure 7.**
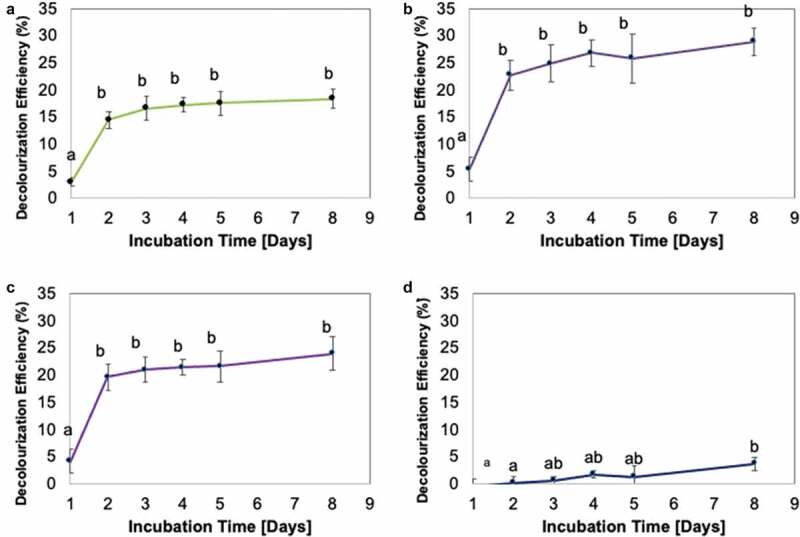
Decolourization Efficiency (DE, %) for TPM-dyes (A) malachite green, (B) methyl violet, (C) crystal violet and (D) cotton blue, over 8 days. Means with the same letters and caption are not significantly different at HSD_(0.05)_. Error bars indicate Standard error of mean (±SEM).

The dye decolourisation rate by Fe-NPs revealed that dyes (except CB) were typically rapidly decolourised within the first 24 h (1 day) upon treatment with Fe-NPs (Figure 7). The decolourisation activities thereafter revealed no significant changes. This implied possible saturation of binding sites on Fe-NPs. Similar observations have been reported for biogenically synthesised Fe-NPs when applied to other dyes, such as methylene blue and methyl orange (Shahwan et al. 2011), as well as for the TPM dye MG (Weng et al. 2013). Comparatively, the results from this study (18.3% decolourisation of MG) revealed a lower decolourisation activity compared to results by Weng et al. (2013) who reported 91% decolourisation of MG after 60 min with the same dye concentration (100 mg/L). This may be ascribed to Weng et al. (2013) using higher quantity of Fe-NPs (48.8 mg Fe-NPs) against a lower volume of dye solution (8 mL). As such, we theorise that if ~50 mg Fe-NPs from our study is used, the decolourisation efficiency achieved may be comparable to the 91% observed in Weng et al. (2013). The other possible factor is perhaps the different biological extracts used. The Fe-NPs by Weng et al. (2013) were synthesised using extracts of green tea, high in polyphenols and phenolic compounds. In this study, the fungal extracts of *F. proliferatum* used may not have the same chemical profile as extracts from green tea; hence, the differences in quality of Fe-NPs produced and the subsequent decolourisation efficiency.

**Figure 8.**
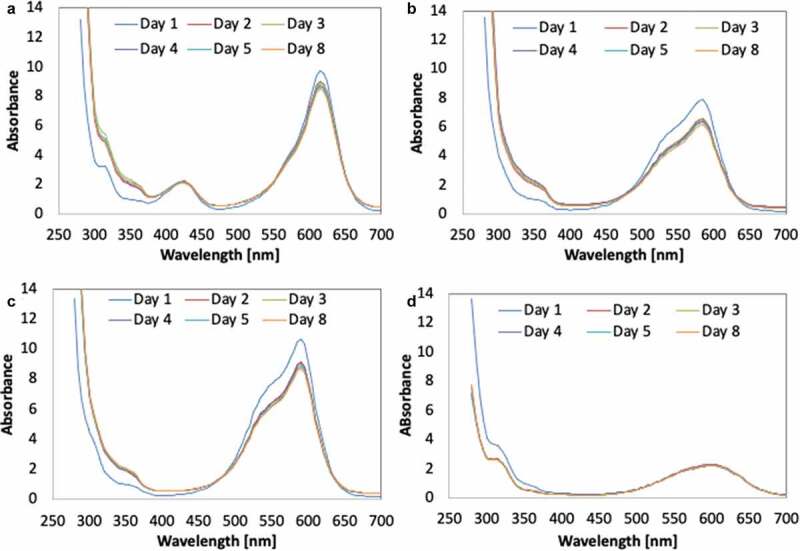
UV-vis spectra of TPM-dyes (A) malachite green, (B) methyl violet, (C) crystal violet and (D) cotton blue, upon treatment with Fe-NPs.

The decolourisation activities suggested that the mechanism involved in dye decolourisation demonstrated by biogenically synthesised Fe-NPs was most likely biosorption. The potential of dye decolourisation through biosorption is limited to the surface area of Fe-NPs and to the amount of Fe-NPs used. The TPM dyes bind to the surface of the Fe-NPs. And with most binding sites saturated, no further binding (biosorption) could occur, leading to maximum biosorption and no gradual increase in decolourisation efficiency (Figure 7). The absence of biodegradation as a means to decolourise the TPM dyes is validated by the UV-vis spectra of each dye. The observation by time interval showed that as the process of decolourisation occurs, the peaks for each respective dye were still detectable (Figure 8). This suggested that the integrity of the dye structure is still present within the dye solution. If biodegradation occurs, peaks would have diminished over the weeks. Evidently, the Fe-NPs could only perform biosorption to remove/decolourise TPM dyes.

## Conclusions

This study is among the first few that documents the successful biogenic synthesis of iron nanoparticles (Fe-NPs) using extracts from endophytic fungi (*Fusarium proliferatum*). The biologically synthesised Fe-NPs have characteristics and properties that are valuable and acceptable as per NP standards. Fe-NPs were similar in size and shape, and the Fe-NPs inherited active functional groups on their surface attributed to the biological components in the extract. The findings here offer alternative synthesis pathways for valuable metal NPs, which is relatively easy and rapid to produce and is more sustainable and environmental friendly. The synthesised Fe-NPs showed the potential to remove triphenylmethane dyes (i.e. malachite green, methyl violet and crystal violet), which are new findings for biogenically synthesised Fe-NPs.
